# Central Composite Design Optimization of Zinc Removal from Contaminated Soil, Using Citric Acid as Biodegradable Chelant

**DOI:** 10.1038/s41598-018-20942-9

**Published:** 2018-02-08

**Authors:** Farrokh Asadzadeh, Mahdi Maleki-Kaklar, Nooshin Soiltanalinejad, Farzin Shabani

**Affiliations:** 1 Department of Soil Science, Urmia University, Urmia, Iran; 2 Department of Chemical Engineering, University of Zanjn, Zanjan, Iran; 3 School of Environmental and Rural Science, University of New England, Armidale, NSW 2351 Australia

## Abstract

Citric acid (CA) was evaluated in terms of its efficiency as a biodegradable chelating agent, in removing zinc (Zn) from heavily contaminated soil, using a soil washing process. To determine preliminary ranges of variables in the washing process, single factor experiments were carried out with different CA concentrations, pH levels and washing times. Optimization of batch washing conditions followed using a response surface methodology (RSM) based central composite design (CCD) approach. CCD predicted values and experimental results showed strong agreement, with an R^2^ value of 0.966. Maximum removal of 92.8% occurred with a CA concentration of 167.6 mM, pH of 4.43, and washing time of 30 min as optimal variable values. A leaching column experiment followed, to examine the efficiency of the optimum conditions established by the CCD model. A comparison of two soil washing techniques indicated that the removal efficiency rate of the column experiment (85.8%) closely matching that of the batch experiment (92.8%). The methodology supporting the research experimentation for optimizing Zn removal may be useful in the design of protocols for practical engineering soil decontamination applications

## Introduction

Soil contamination by heavy metals has become a global concern, due to the threat to ecosystems and human health of their high toxicity levels^1,2^. In agricultural areas, heavy metal contamination, even if not associated with specific health hazards, may reduce economic output^2^. The United States Environmental Protection Agency (USEPA) has classified Zinc (Zn) as a harmful heavy metal, and placed it on the list of priority pollutants^3^.

Soils polluted with heavy metals can be decontaminated through a variety of methods of remediation, such as stabilization, solidification, electroremediation and phytoremediation^4^. Among the techniques of remediation, the simplicity of chemical soil washing, its low cost, short duration and high efficiency, makes it a practical option for metal removal^5–7^. The selection of a viable solution is a fundamental determinant of the level of efficiency of extraction in the washing process^3,6,8^. Environmental factors, and the cost and availability of the extraction solution, are other important considerations in selecting a washing reagent^3^. Due to its superior capacity to complex with a number of heavy metal ions, the chelating agent ethylenediaminetetra acetic acid (EDTA) has frequently been used in polluted soil treatments^9^. However, EDTA usage has been restricted due to its poor level of biodegradability^8^ and it has been replaced by natural biodegradable chelants, citric acid (CA) being the most prominent^10,11^, due to its minimal impact on the physical and chemical properties of soil^12^.

Optimum soil washing conditions are prerequisite, particularly in the case of scaled up applications, to minimise costs and maximise Zn removal efficiency. Classical optimization, in which only one factor is changed at a time in order to measure its effect, is time consuming and requires many experiments. In practice, it overlooks, or disregards, the interactive effects between individual components. Response surface methodology (RSM), in which effective parameters are optimised simultaneously, overcomes the deficiencies of single factor optimization^13,14^. Using RSM substantially reduces the number of experiments necessary to predict the conditions for best performance^15,16^. Furthermore, modelling of the process refines the interpretation of complex phenomena and provides a basis for process scaling^17^.

Regarding the advantages of RSM, it has been used successfully in the modelling and optimization of soil washing conditions, for the efficient removal of Zn from severely contaminated soil. Thus, our principal research objective set out to investigate the process of removing Zn from highly contaminated soil, using CA as an ecologically benign, biodegradable chelant in a batch washing process. In order to predict optimal conditions for Zn removal, we developed a quadratic polynomial model, using a central composite design (CCD) based on RSM. Thereafter, the optimized batch washing conditions were applied to a column washing experiment, to compare the efficiency of the two washing methods.

## Materials and Methods

### Soil sampling

A contaminated soil sample was collected from the topsoil layer (0–20 cm) around the Angouran zinc mine in Zanjan Province, Iran (Lat. 47°15′17″, Long 35°44′37″). The sample was air dried, homogenized, sifted through a 2 mm nylon mesh sieve for the removal of larger particles, and stored at room temperature in plastic containers.

The following properties of the sample soil were analysed: texture, pH, electrical conductivity (EC), cation exchange capacity (CEC), soil organic matter (SOM) and calcium carbonate equivalent (CCE). To determine texture, the hydrometer method as described by Gee *et al*.^18^ was used. SOM was determined by dichromate oxidation, and soil pH and electrical conductivity (EC) were determined in a soil-solution ratio of 1:5 using pH and electrical conductivity meter^19^. CEC was measured by saturating soil with 1 M NH_4_OAc at pH 7, and CCE was determined by the acid neutralization method^19^. The total zinc content was measured using a flame atomic absorption spectrophotometer (Shimadzu AA-7000) based on a soil sample with HNO_3_-HCl-HClO_4_ mixture at a 1:2:2 ratio (v/v/v). Values of these sample physicochemical properties are listed in Table 1.

**Table 1:** Physicochemical characteristics of polluted soil before washing.

Characteristics	Unit	Value
EC	dS.m^−1^	0.7
pH	—	7.8
Soil texture	—	Clay loam
Sand	%	34
Silt		37
Clay		29
SOM		1.03
CCE		21
CEC	cmol_+_.kg^−1^	17
Total Zn	mg.kg^−1^	5657

The sample was calcareous (CCE = 21%), slightly alkali (pH = 7.8), with texture classified as clay loam. The total Zn concentration of 5657 mg.kg^−1^ surpassed the level permitted in soil used primarily for the agricultural production of food crops.

### Preliminary soil washing experiments

The preliminary ranges of washing variables, the CA concentration, pH of solution and washing time were determined in a sequence of single factor experiments, using a soil-solution ratio of 1:10 (w/v) in 100 mL acid-rinsed polycarbonate plastic bottles. For each run, 5 grams of the contaminated sample was added to 50 mL of the washing reagent.

To investigate the effect of CA concentration on Zn removal, a range of concentrations of CA from 5 to 400 mM were tested. The pH test was based on 200 mM CA at a range of pH values from 2.0–10.0, altered with diluted HNO_3_ and/or NaOH solution. A 200 mM CA solution was also prepared to measure the effects of washing time, ranging from 15 to 300 min, on removal efficiency.

The suspensions in each test were mixed using a mechanical shaker, running at a constant 150 rpm for the predefined time. Following the shaking, suspensions were centrifuged for 10 min, at 4000 rpm, and then filtered through a Whatman (0.45 µm) filter membrane. The supernatant Zn concentration was measured using a flame atomic absorption spectrophotometer (AAS). All of these tests were conducted in duplicate.

### Experimental design and optimization

The RSM based central composite design (CCD) with independent variables was followed to create the optimum synergy of and to check the response patterns. The specific parameters whose ranges had been established in the preliminary testing, CA concentration (20–200 mM), pH (4–8), and washing time (30–150 min) were thus optimized (Table 2).

**Table 2:** Experimental range and level of independent variables.

Independent Variables	Symbol	Range and level				
		−α	−1	0	+1	+α
pH	X_1_	4	4.81	6	7.19	8
Concentration (mM)	X_2_	20	56.48	110	163.51	200
Time (min)	X_3_	30	54.32	90	125.68	150

For the CCD, the selected experimental points included eight cubic points, six axial points (α = ±1.68), and six replicates at the centre point (α = 0).

Optimum variable values were calculated from the experimental response and coded at five levels from −1.68, −1, 0, +1, and +1.68 as defined by Eq. (1).

1 $${{\rm{x}}}_{{\rm{i}}}=\frac{{{\rm{X}}}_{{\rm{i}}}-{{\rm{X}}}_{0}}{{\rm{\Delta }}{{\rm{X}}}_{{\rm{i}}}}$$

where, x_i_ denotes the coded value of variable X_i_, X_0_ the actual value of X_i_ at the centre point, and ΔX_i_ the increment.

CCD model was designed to fit the second order polynomial model, using a multiple regression program according to Eq. (2).

2 $${\rm{Y}}={{\rm{\beta }}}_{0}+\,\sum _{{\rm{i}}=1}^{{\rm{k}}}{{\rm{\beta }}}_{{\rm{i}}}{{\rm{x}}}_{{\rm{i}}}+\sum _{{\rm{i}}=1}^{{\rm{k}}}{{\rm{\beta }}}_{{\rm{ii}}}{{\rm{x}}}_{{\rm{ii}}}^{2}+\sum _{{\rm{i}}=1}^{{\rm{k}}-1}\sum _{{\rm{j}}=2}^{{\rm{k}}}{{\rm{\beta }}}_{{\rm{ij}}}{{\rm{x}}}_{{\rm{i}}}{{\rm{x}}}_{{\rm{j}}}+{\rm{\varepsilon }}\quad \quad {\rm{i}}\ne {\rm{j}}$$

where Y represents the variable of response (percentage Zn removal), X_i_ and X_j_ independent coded variables, and β_0_, β_i_, β_ii_, β_ij_ the intercept term, linear, quadratic and interaction effects, respectively^16,20^. Random error (ɛ) expresses the measure of difference between observed and predicted values. To give greater insight into the CCD results, Pareto analysis was used to calculate the percentage effect of each independent variable (P_i_) on the removal of Zn^21^ (Eq. 3):

3 $${{\rm{P}}}_{{\rm{i}}}=(\frac{{{\rm{\beta }}}_{{\rm{i}}}^{2}}{\sum {{\rm{\beta }}}_{{\rm{i}}}^{2}})100\quad {\rm{i}}\ne 0$$

Minitab 14 statistical package (MINITAB Inc., PA, USA) was used for the statistical analysis of the results.

## Results and Discussion

### Preliminary washing experiment

**Figure 1:**
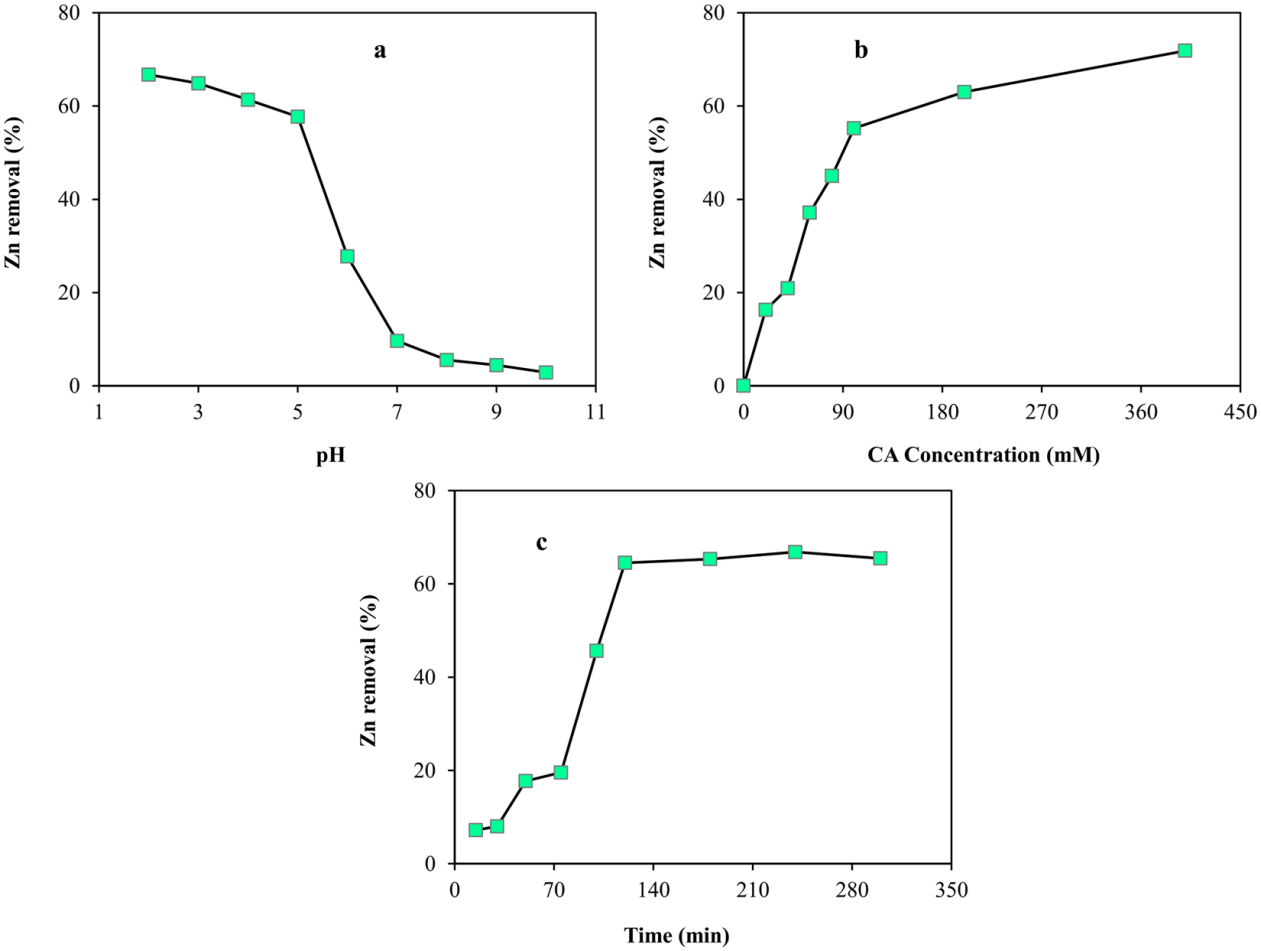
Preliminary single factor washing experiments; Zn removal as a function of (**a**) pH, (**b**) CA concentration, and (**c**) washing time.

In the preliminary testing, CA removal efficiency followed the trend of decreasing Zn removal with increasing pH (Fig. 1a). Results indicated that when pH was decreased from 8 to 4, Zn removal increased considerably from 5.5% to 61.3%, while remaining almost constant for lower pH values. This implies that acidic conditions, rather than alkaline, are preferable for Zn removal. Previous research findings have indicated that the pH of the solution plays a major role in the removal efficiency of heavy metals from contaminated soils^6,8,22^. Accordingly, the behaviour of metal-chelant complexes, solubility of the zinc hydroxides, competition between H^+^ and the Zn^2+^ for sorption sites on soil particles, as well as the surface charge of the soil colloids, is directly controlled by pH. A rise in pH of the CA solution increases the cationic heavy metal adsorption on the soil surface, inner sphere complexion, and may also increase the probability of precipitation reactions ^6,23^.

Figure 1(b) illustrates the effect of CA concentration on Zn removal efficiency, at a fixed washing time of 120 minutes. The zinc removal was less than 0.5% when distilled water was applied as a washing solution, which might be due to the very low solubility of zinc compounds in the water. Further, the zinc removal increased considerably for concentrations of CA up to 100 mM, with a slower increase at higher concentrations, which can be ascribed to the high efficiency of CA as a chelating agent. Citric acid forms a square planar complex with heavy metals, through binding to the citrate anions with cations. Huang *et al*.^24^ also noted the increase in heavy metals removal with increasing CA concentrations, in the range lower than the 200 mM. This could be explained by the high stability content (Log K_ML_ = 6.1) for Zn-CA complexes^25^.

Washing time is another influential factor in remediation of heavy metals from contaminated soil, in that the process of desorption is a kinetic equilibrium. Figure 1c shows the kinetics of Zn removal by CA (concentration = 100 mM; pH = 4.0) for washing times ranging from 15 to 300 min. Zn removal efficiency increased within the time range of 30 to 120 min, before approaching a relatively constant level, indicating that soil washing with CA is a time dependent process. This observation shows a strong agreement with other studies, regarding washing time^6,7,22,26^. The initial rapid increases in Zn removal, followed by a levelling, may be due to the fact that the chelation process reaches equilibrium at a certain length of washing time.

From the preliminary results, the range of independent variables, as listed in section 2.3 (Table 1), was selected for the RSM based central composite design (CCD). In the following section, the results of Zn removal optimization, using RSM based CCD are presented.

### CCD modelling

The CCD experiment design for Zn removal, based on three independent factors with a five- level structure, is shown in Table 3. The broad range of Zn removal (from 5.35 to 79.11%) demonstrates the necessity to optimize washing conditions. The quadratic polynomial equation (Eq. 4), obtained from experimental results which expressed as coded units, defines the relationship between the independent variables (CA concentration, pH, and washing time) and response (percentage Zn removal).

4 $$\begin{array}{c}{\rm{Y}}=\,28.61-23.84{{\rm{x}}}_{1}+11.49{{\rm{x}}}_{2}-1.76{{\rm{x}}}_{3}+4.96{{\rm{x}}}_{1}^{2}\\ \quad \,\,\,\,+\,2.44{{\rm{x}}}_{2}^{2}-1.53{{\rm{x}}}_{3}^{2}-7.38{{\rm{x}}}_{1}{{\rm{x}}}_{2}+5.56{{\rm{x}}}_{1}{{\rm{x}}}_{3}+5.72{{\rm{x}}}_{2}{{\rm{x}}}_{3}\end{array}$$

**Table 3:** The three factor CCD matrix in coded units.

Run	Independent Variable			Zn Removal (%)	
	pH	CA Conc. (mM)	Time (min)	Measured	Predicted
1	1.00	1.00	1.00	15.55	24.28
2	0.00	0.00	0.00	28.64	28.61
3	0.00	0.00	1.68	27.93	21.33
4	0.00	0.00	0.00	28.64	28.61
5	1.68	0.00	0.00	5.35	2.55
6	0.00	−1.68	0.00	15.82	16.18
7	0.00	1.68	0.00	54.36	54.84
8	1.00	−1.00	−1.00	5.57	8.45
9	−1.00	1.00	−1.00	78.66	78.80
10	−1.68	0.00	0.00	79.11	82.75
11	−1.00	−1.00	−1.00	61.85	52.50
12	−1.00	1.00	1.00	79.11	75.61
13	0.00	0.00	−1.68	19.80	27.25
14	0.00	0.00	0.00	28.64	28.61
15	1.00	−1.00	1.00	5.35	4.60
16	0.00	0.00	0.00	28.64	28.61
17	0.00	0.00	0.00	28.64	28.61
18	−1.00	−1.00	1.00	20.33	26.42
19	0.00	0.00	0.00	28.64	28.61
20	1.00	1.00	−1.00	11.93	5.24

The CCD predicted Zn removal values, as determined by the quadratic polynomial model (Eq. 4), are shown in Table 3, and display generally strong agreement with the measured values of Zn removal derived from the experiment results. The model validation resulted in a coefficient of determination (R^2^) of 0.966, and root mean square error (RMSE) of 4.4% (see supporting information, Fig. S1) in agreement of empirical and predicted values. Thus, 3.4% of the variation in Zn removal is not supported by the CCD model.

### Analysis of variance (ANOVA)

Table 4 displays the ANOVA results for the fitting of the quadratic model. The table indicates that in both the linear and square parameters, the effects of the prominent variables are significant on response, with p-values < 0.05. Similarly, the response levels of the interactions of independent variables are also significant with p-values < 0.05.

**Table 4:** ANOVA results for Zn removal.

Model parts	Source	Zn Removal			
		DF	Mean square	F-Value	P-Value
—	Model	9	1226.12	31.66	0.00
—	Linear	3	3202.99	82.71	0.00
Linear	pH	1	7762.7	200.46	0.00
	CA	1	1804.08	46.59	0.00
	Time	1	42.21	1.09	0.32
—	Square	3	160.4	4.14	0.04
Square	pH*pH	1	355.16	9.17	0.01
	CA*CA	1	85.86	2.22	0.17
	Time*Time	1	33.63	0.87	0.37
—	Interaction	3	314.97	8.13	0.01
Interaction	pH*CA	1	435.58	11.25	0.01
	pH*Time	1	247.19	6.38	0.03
	CA*Time	1	262.15	6.77	0.03

R-sq = 0.9661 R-sq(adj) = 0.9356.

In the ANOVA Table, the model F-value of 31.66 is notably greater than the tabulated F-distribution value of 3.02 at 95% significance, indicating that the model is conclusively efficient in predicting experimental results. Table 5 lists the regression coefficients, t-values, and p-values for the linear, quadratic, and effects of variable interactions, at a significance level of 95%. The significance of each term of the model was verified by means of the associated p-value.

**Table 5:** Estimated regression coefficients, t-values and p-values.

Term	Zn Removal		
	Coefficient	T-Value	P-Value
β_0_	28.61	11.27	0.00
β_1_	−23.84	−14.16	0.00
β_2_	11.49	6.83	0.00
β_3_	−1.76	−1.04	0.32
β_11_	4.96	3.03	0.01
β_22_	2.44	1.49	0.17
β_33_	−1.53	−0.93	0.37
β_12_	−7.38	−3.35	0.01
β_13_	5.56	2.53	0.03
β_23_	5.72	2.60	0.03

The liner effect of pH, CA concentration and the second order effect of pH are significant model terms on Zn removal (p-value < 0.05). Furthermore, interaction effects of all three independent variables (pH, CA concentration, and washing time) are also significant.

**Figure 2:**
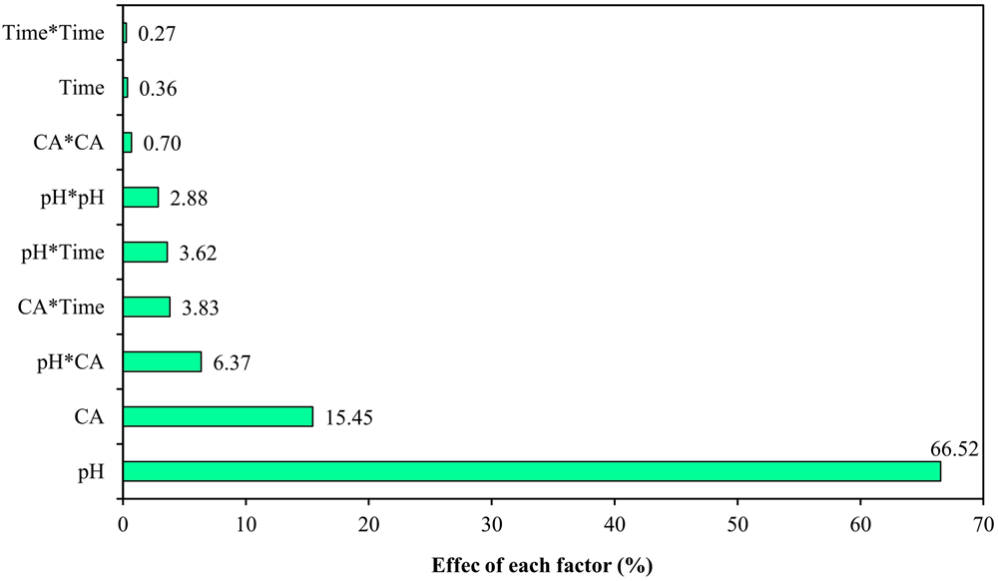
Graph of Pareto analysis ranking effectiveness of individual factors on removal response.

Pareto analysis ranks the effect of variables on response (Eq. 3). The analysis indicated that the order of effective variables on Zn removal was pH (66.52%) > CA concentration (15.45%) > pH and CA concentration interaction (6.37%) > washing time and CA concentration interaction (3.83%)> washing time and pH interaction (3.62%) > quadratic effect of pH (2.88%). These six terms accounted for 98.67% of cumulative effects on Zn removal (Fig. 2).

To produce the optimised model for Zn removal, the final quadratic response surface model (Eq. 5), expressed as coded units, represents the CCD model with the removal of insignificant terms (p-value > 0.05).

5 $$\begin{array}{c}{\rm{Y}}=28.61-23.84{{\rm{x}}}_{1}+11.49{{\rm{x}}}_{2}+4.96{{\rm{x}}}_{1}^{2}\\ \quad \,\,\,-\,7.38{{\rm{x}}}_{1}{{\rm{x}}}_{2}+5.56{{\rm{x}}}_{1}{{\rm{x}}}_{3}+5.72{{\rm{x}}}_{2}{{\rm{x}}}_{3}\end{array}$$

### Effect of independent variables

**Figure 3:**
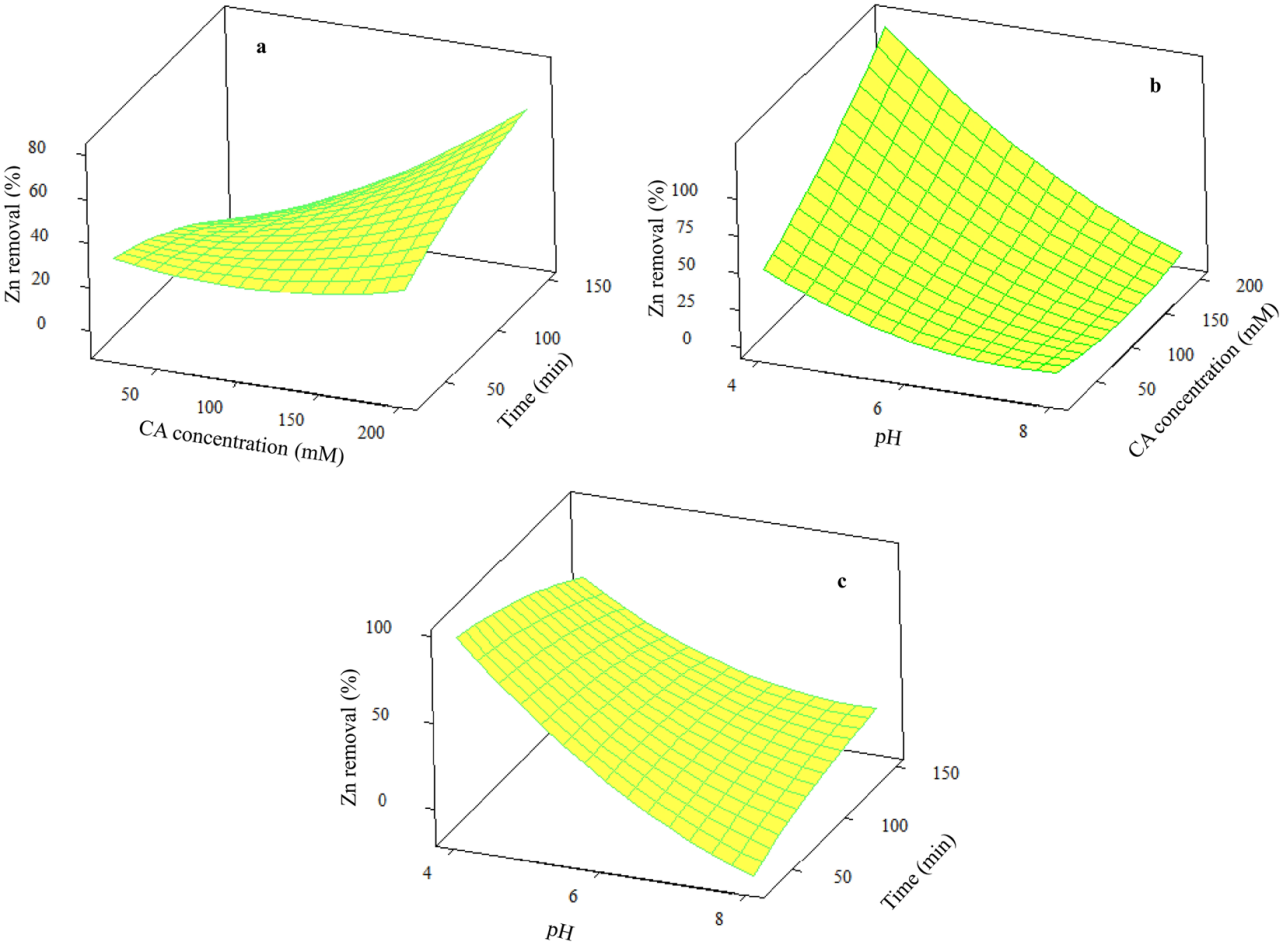
3D surface plots of Zn removal (%) as a function of: (**a**) CA concentration (mM) and time (min); (**b**) CA concentration (mM) and pH; (**c**) pH and time (min).

The independent variable interactions are illustrated by 3D surface plots, derived from the quadratic polynomial model, in Fig. 3(a–c). In each 3D surface plot, two variables are altered, while the third variable is maintained at a constant value of zero coded level. The shape of these plots supports an interpretation of the level and quality of the interactions of the independent variables.

Figure 3b shows the trend of Zn removal against upon coinciding variation of washing time and CA concentration at constant pH of 6. As expected, the removal efficiency increased proportionate to the CA concentration. Removal was significantly enhanced as the washing time increased from 30 to 150 min. An increased CA concentration promotes the reaction of the heavy metal ion-ligand complex to move toward the direction of chelate formation^6,7^. Similarly, Ren *et al*.^5^ reported a significant increase in the efficiency of Zn removal from sewage sludge, with increases of the CA concentration over the range of 200–1000 mM.

The combined effect of CA concentration level and pH value on Zn removal efficiency, at a fixed washing time of 90 min, is presented in Fig. 3b. Here it can be seen that the efficiency of Zn removal depends strongly on CA concentration and pH, with a higher CA concentration increasing efficiency, but a higher pH having a negative effect. The pH effect was more notable at higher CA concentrations, and thus maximum Zn removal occurred at lower pH values, with increased CA concentration. As the pH decreases, the surface of the soil colloids commonly acquires a positive charge, due to the protonation of functional groups. This promotes heavy metals desorption from the soil surface. Additionally, hydrogen ions are weak competitive cations which can replace the adsorbed heavy metals via the cation exchange mechanism^27^.

The effect of simultaneous variations of pH and time is illustrated in Fig. 3c. The CCD model was applied with pH increasing from 4 to 8, and washing time from 30 to 150 min. Figure 3c illustrates that removal efficiency is enhanced by decreasing the pH, but the effect of time appears to be more intricate. According to the results of the CCD model (Table 4), Zn removal is not directly influenced by washing time, the effect of which is only significant in terms of interactions. Likewise, Tejowulan and Hendershot^28^ demonstrated that the heavy metal removal rate was dependent on chelant concentration, and not on washing time.

### Determination of optimal conditions

Optimal values of the independent variables, CA concentration, pH and washing time, as given in Table 6, to achieve a Zn removal efficiency of 95%, were obtained by a response optimizer. For validation, a confirmation experiment was conducted, with four replicates at the optimum conditions predicted by CCD model. Zn removal efficiency obtained from the experiments at optimal condition (92.80 ± 4.84%) showed a strong agreement with that of the predictive model value (95%). The results suggest that the use of CCD for optimization of soil washing conditions, to attain the maximum efficiency of Zn removal, has proven successful.

**Table 6:** Optimal values of the independent variables, experimental, and RSM predicted Zn removal.

pH	CA concentration (mM)	Washing time (min)	The Zn removal (%)	
			Measured ± SD*	Predicted
4.43	167.60	30.00	92.80 ± 4.84	95.00

^*^SD: Standard deviation of the 4 replicates.

### Leaching column experiment

To compare the efficiency of batch and column washing techniques, a leaching column experiment was executed at the optimum CCD batch washing values of CA concentration (167.6 mM) and pH (4.43). The leaching column consisted of PVC tube (20 cm length, 5 cm diameter), with the contaminated soil sample positioned at a height of 10 cm by uniform tapping with a wooden rod to create a constant bulk density of 1.3 g.cm^−3^ (total porosity ≈ 50%). The top and bottom of the column were filled with an approximately 1 cm filter gravel layer. Wathman NO. 47 filter paper was used to preclude the loss of soil particles, from the bottom of the each column, during leaching.

The cumulative leaching of the Zn is depicted as a function of pore volume in Fig. S2 (see supporting information). The leaching column experiment indicated that Zn removal by CA solution (167.6 mM, pH = 4.43) is a two-step process, commencing with a fast desorption within the initial pore volumes, followed by a slower desorption occurring over the subsequent pore volumes. The more pore volumes that are added, the more Zn may be removed. From the first 15 pore volumes, equal to a 1:6 (w/v) soil to water ratio, 76.24% of the final total Zn was leached from the soil. Thereafter, the removal efficiency increased slightly, culminating in 85.8% at the 26^th^ pore volume (soil water ratio of 1:10 (w/v) similar to the batch experiments). Further soil washing had little effect on the overall Zn removal, which is consistent with the findings of Lo *et al*.^29^ and Elmaslar-Özbaş and Balkaya^30^, in regard to leaching.

The comparison of two soil washing techniques with CA, batch washing vs. column washing, indicated that the removal efficiency for the column experiment (85.8%) is comparable with those of the batch experiment (92.8%). It is noteworthy that although Zn removal by batch washing experiment is less time consuming, and slightly more efficient than column washing, the latter technique has some benefits. Column leaching minimizes worker exposure to contamination. Additionally, it is more practical and has the potential for the economical treatment of relatively large amounts of soil. During the column washing procedure the soil structure remains undisturbed, unlike the batch techniques^4^. However, it should be noted that the column leaching technique may be very difficult to apply in low permeable soils^31^.

## Conclusions

Zn removal from contaminated soil, by CA as a biodegradable chelant, was modelled and optimized by the response surface methodology (RSM) based central composite design (CCD). The following conclusions can be drawn from this research:

- RSM based CCD is a promising tool (R^2^ = 96.6%) for modelling and optimizing Zn removal from contaminated soil by CA.
- The optimum values of pH, CA concentration, and process time were estimated at 4.43, 167.6 mM, and 30 min, respectively, with 95% Zn removal as target efficiency.
- It was found that the pH and CA concentration with a percentage importance of 66.52% and 15.45% respectively, are the most significant parameters in the Zn removal process.
- The optimal values for pH and CA concentration from the batch washing experiments could be successfully translated to column washing, and the removal yields were efficient and comparable under both techniques.

## Electronic supplementary material

Supplementary Information
