# Supplementary material for: Central Composite Design Optimization of Zinc Removal from Contaminated Soil, Using Citric Acid as Biodegradable Chelant
Source: Sci Rep. 2018 Feb 8;8:2633. doi: 10.1038/s41598-018-20942-9 (PMC5805782; doi:10.1038/s41598-018-20942-9)
Supplement: Supplementary file 1 — Supplementary Information [file 41598_2018_20942_MOESM1_ESM.docx]

**Supplementary Information for CENTRAL COMPOSITE DESIGN OPTIMIZATION OF ZINC REMOVAL FROM CONTAMINATED SOIL, USING CITRIC ACID AS BIODEGRADABLE CHELANT**

Farrokh Asadzadeh^1^*, Mahdi Maleki-Kaklar^2^, Farzin Shabani^3^, Nooshin Soiltanalinejad^1^

^1^ Department of Soil Science, Urmia University, Urmia, Iran

^2^ Department of Chemical Engineering, University of Zanjn, Zanjan, Iran

^3^School of Environmental and Rural Science, University of New England, Armidale NSW 2351 Australia

Author correspondence E-mail: f.asadzadeh@urmia.ac.ir; Phone: +98 44 32752740; Fax: +98 44 32753172

Fig. S1. Comparison between the experimental results and predicted values for Zn removal by the tested CCD model

Fig. S2. Cumulative removal the Zn as a function of the pore volume
